# Acquired ROS1 fusion and iruplinalkib response in advanced NSCLC after multiple lines of systematic therapy: a case report

**DOI:** 10.3389/fonc.2025.1571512

**Published:** 2025-04-30

**Authors:** Jiarui Liu, Zhichao Jiao, Jun Zhou, Yuan Yuan, Qiguang Li, Wei Zhou, Shuai Zhang, Shuping Yang

**Affiliations:** ^1^ Department of Oncology, Shandong Provincial Hospital Affiliated to Shandong First Medical University, Jinan, Shandong, China; ^2^ The Clinical Medical College, Shandong First Medical University (Shandong Academy of Medicine), Jinan, Shandong, China; ^3^ Department of Radiology, Shandong Provincial Hospital Affiliated to Shandong First Medical University, Jinan, Shandong, China

**Keywords:** case report, ROS1 fusion, iruplinalkib, NSCLC, ALK TKI

## Abstract

This is the first report of a patient with lung cancer whose primary focus was the upper lobe of the left lung combined with multiple metastases in both lungs, initially diagnosed as a non-driver gene mutation, who subsequently developed SDC4-ROS1 fusion after multiple lines of systemic therapy. When diagnosis, a needle biopsy of the primary focus revealed no driver gene mutation and low PD-L1 expression (TPS < 1%, CPS 3). From November 2022 to December 2023, the patient received sequential chemotherapy-based systemic therapy including anti-angiogenesis treatment, concurrent chemoradiation and combined immunotherapy as determined by the clinician based on the initial evaluation. In December 2023, a needle biopsy of a metastasis in the left lower lobe of the lung showed a positive SDC4-ROS1 fusion. Subsequent treatment with the oral ALK TKI iruplinalkib was initiated based on the patient’s preference, which exhibited a promising response over the next 2 months.

## Introduction

A receptor tyrosine kinase is encoded by the proto-oncogene ROS1, and somatic chromosomal fusions involving ROS1 result in chimeric oncoproteins that fuel various cancers in both adults and children (1). Tyrosine kinase inhibitors (TKIs) targeting ROS1 have therapeutic activity for these cancers, while some ALK TKIs such as repotrectinib are also used in the treatment of patients with ROS1 fusion due to the 70% homology between the kinase structural domains of ALK and ROS1 (1, 2). ROS1 fusions are relatively rare drivers in non-small cell lung cancer (NSCLC), occurring in 1-2% of cases. Previous evidence suggests that first-line chemotherapy may induce genomic alterations in driver oncogenes such as EGFR, leading to the development of acquired mutations and potential benefits from targeted therapy. Here, we present a case of advanced NSCLC initially negative for ROS1 fusion at diagnosis, but subsequently identified as ROS1 fusion-positive. Several previous studies confirmed that ROS1 is the gene most likely to be missed in DNA NGS assays, with the potential for false-negative results (3), therefore, we performed a duplicate NGS assay for molecular profiling on the same specimen and obtained the same negative results. Ultimately, the patient derives clinical benefit from a novel ALK TKI after undergoing multiple lines of systemic therapy.

## Case report

**Figure 1:**
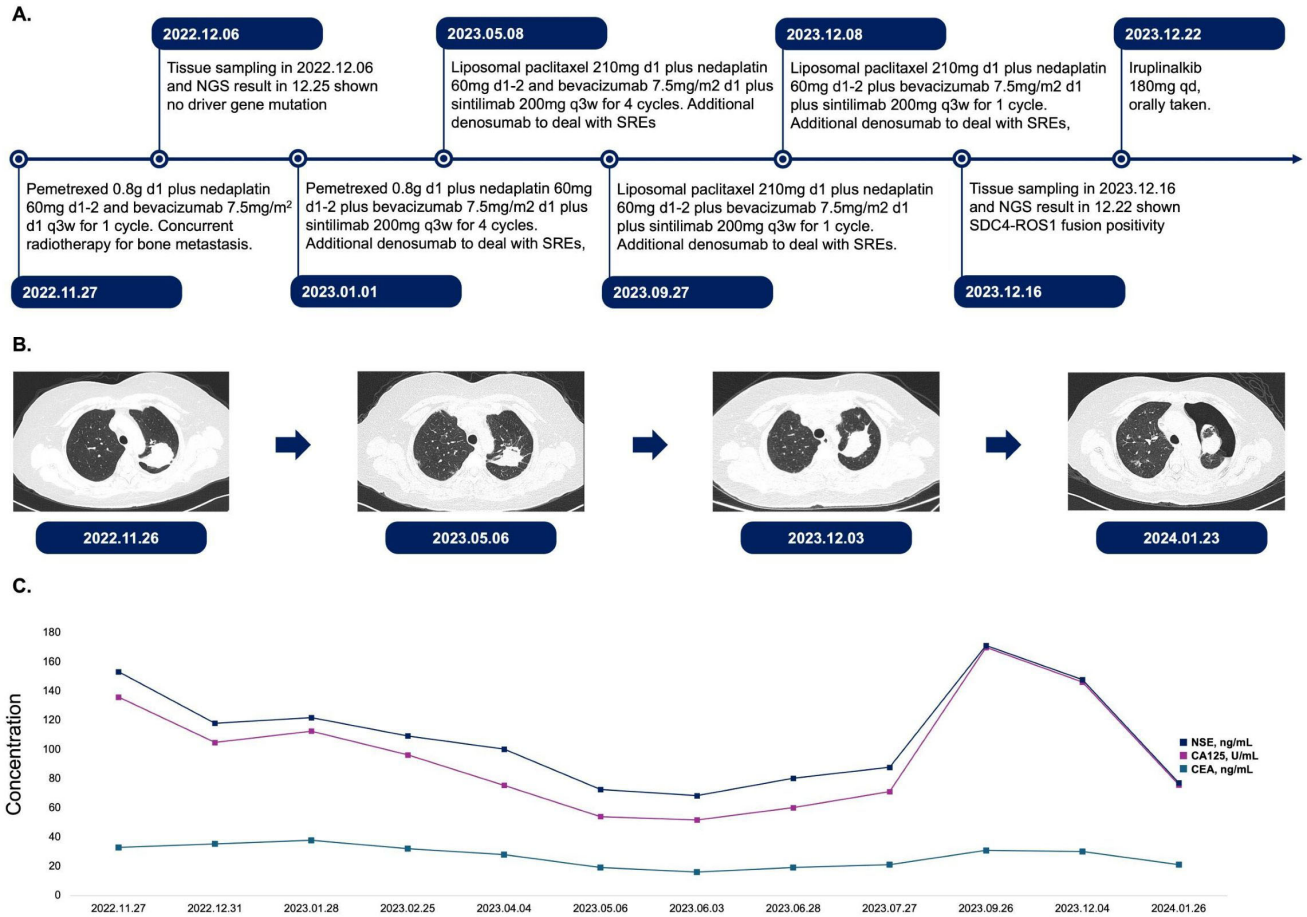
Treatment course, CT examination, and tumor biomarker expression in this patient. **(A)**. Timeline of treatment regimens from November 2022 to December 2023. The patient received various combinations of chemotherapy agents, targeted therapies, and immunotherapies. Treatment cycles and dosages are indicated for each regimen. SREs were managed with denosumab. **(B)**. Serial chest CT images demonstrating the evolution of the primary lesion in upper lobe of left lung. Images are shown from November 2022 to January 2024, corresponding to different stages of treatment. **(C)**. Longitudinal assessment of tumor biomarkers NSE, CA125, and CEA from November 2022 to January 2024. The graph illustrates fluctuations in biomarker levels for treatment. d, day; q3w, every 3 weeks; qd, once daily; SREs, skeletal-related events; NSE, neuron-specific enolase; CA125, cancer antigen 125; CEA, carcinoembryonic antigen.

**Figure 2:**
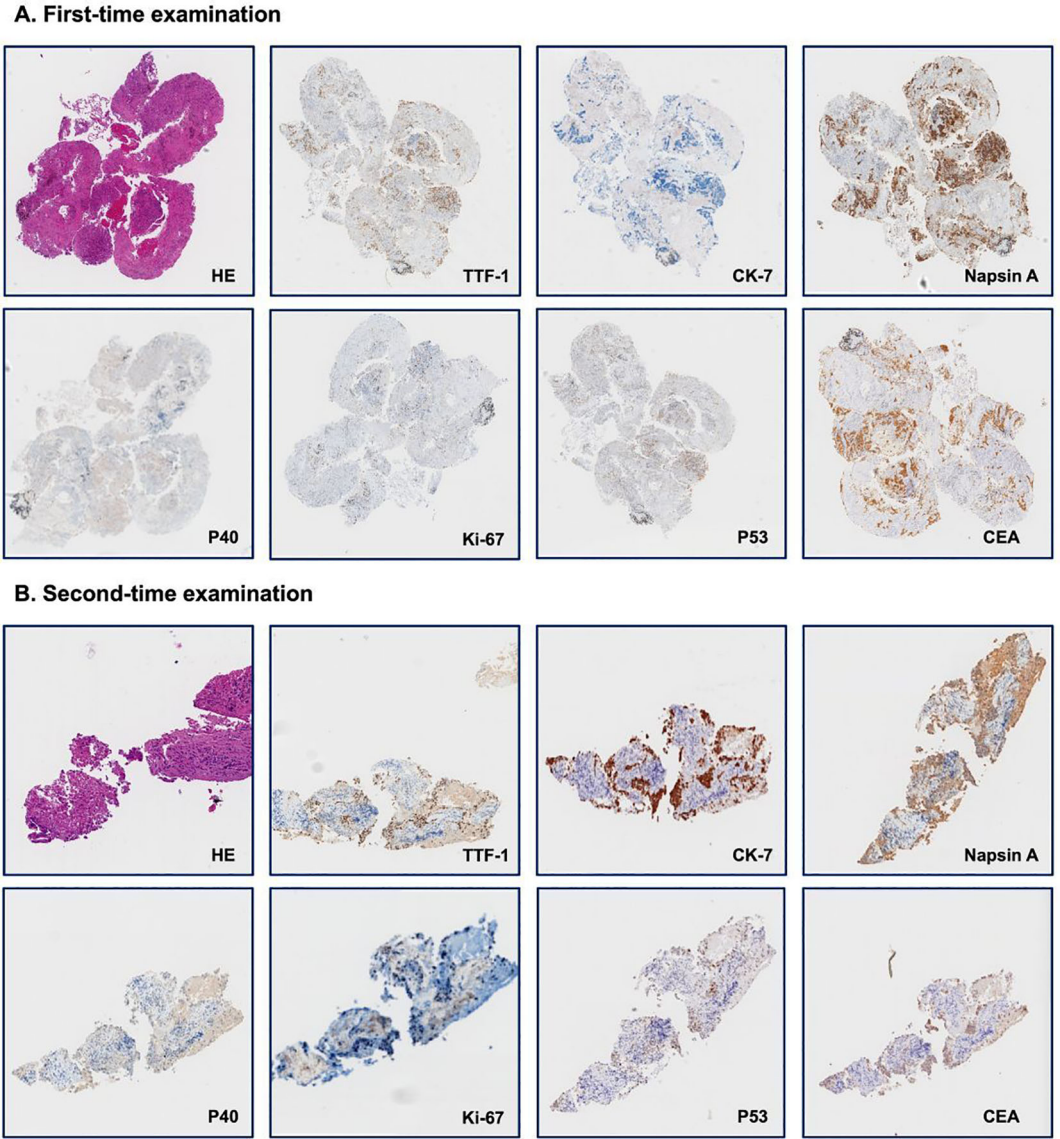
Immunohistology examination of needle biopsy. **(A)**. First-time examination; **(B)**. Second-time examination. Each panel shows staining for hematoxylin and eosin (HE) and seven distinct markers: thyroid transcription factor-1 (TTF-1), cytokeratin-7 (CK-7), Napsin A, P40, Ki-67, P53, and carcinoembryonic antigen (CEA).

A 56-year-old lifelong never-smoker Chinese female presented in November 2022 with a dry cough without apparent precipitang factors, accompanied by shoulder and back pain. The patient had no family history of cancer and no prior underlying conditions, such as hypertension or coronary artery disease. With a primary focus in the upper lobe of the left lung and numerous metastases in both lungs, she was diagnosed with lung adenocarcinoma (cT2N2M1, stage IV). Molecular profiling by NGS on the initial biopsy specimen was negative for potentially actionable driver alterations, including EGFR, ALK, ROS1, BRAF, MET, RET, NTRK, and ERBB2 mutations. Meanwhile, 3 somatic variants with unknown significance (VUS) were detected, including a TP53 missense variant (R282W) and 2 variants in HRR pathway (BRCA2 H523R; RAD51D V66M). The patient also exhibited low PD-L1 expression (TPS <1%, CPS 3) and a low TMB (7.33 Muts/Mb), suggesting limited potential benefit from immunotherapy. The patient had not received any previous treatment, given these findings, the patient received cytotoxic chemotherapy (bevacizumab in combination with pemetrexed and platinum-based chemotherapy), and anti-angiogenic therapy beginning on December 6th, 2022, followed by concurrent chemoradiation and subsequent combined immunotherapy until December 8, 2023, according to the ORIENT-11 trial (4). However, CT imaging revealed further disease progression. The treatment course and histological images are summarized in **Figures 1**, **2**.

Surprisingly, a repeat biopsy of large metastases in the left lower lobe of the lung using NGS on December 16, 2023, detected a newly acquired SDC4-ROS1 fusion (abundance 15.48%), while other potential drivers, such as EGFR, ALK, and BRAF, remained wild-type. Furthermore, none of the three earlier mutations had been found, while ROS1 fusion and another passenger mutation PDGFRB D776E with VAF of 9.5% emerged. The patient was then initiated on the ALK TKI iruplinalkib at a dose of 180 mg daily. Fortunately, the patient’s condition improved significantly at the first review following a month of oral iruplinalkib. On CT images, the maximum diameter of the primary lesion in the upper lobe of the left lung had decreased from 4.9 cm to 4.5 cm. Additionally, the CT report showed that most of the patient’s multiple metastases in both lungs were much smaller than previously. This was particularly true for the metastasis in the lower lobe of the left lung, which was the site of the second biopsy, and the imaging changes we included in the **Supplementary Materials**. Through our follow-up of the patient, on 20 February 2024, the cross-sectional area of the primary lesion was reduced by another 36% compared to the previous examination, and the patient will continue to benefit from the treatment.

## Discussion

ROS1 fusions are well-described oncogenic drivers in NSCLC. They occur in 1-2% of cases and confer sensitivity to ROS1 TKIs like crizotinib.

While uncommon, similar examples of acquired driver oncogenes mutation such as EGFR have been reported (5), particularly after exposure to chemotherapy and other DNA-damaging agents. Similarly, there have been previous case reports of patients with EGFR-mutant advanced NSCLC who developed EML4-ALK fusion mutations after treatment (6). However, to our knowledge, this is the first report that illustrates the potential for ROS1 fusion to emerge later as acquired alterations during treatment of NSCLC. Given the high false-negative rate of ROS1 in DNA NGS assays (3), after performing the NGS test for the second time and obtaining an acquired ROS1 fusion, we repeated the examination of the initial specimen and obtained the same NGS results as the previous time. Similarly, the IHC result on the original specimen was negative. Of course, the location of the needle biopsy and the heterogeneity of the tumor would also be critical considerations, choosing an authoritative and comprehensive NGS assay, as well as repeat testing, is necessary. The mechanisms underlying such acquired ROS1 alterations remain unclear but may relate to tumor heterogeneity, genomic instability and selective pressures induced by cytotoxic and targeted therapies (7). The patient had previously received a platinum-based regimen, and previous studies have shown that cisplatin may increase genomic instability by inducing DNA damage, interfering with repair mechanisms, and epigenetic regulation (8). Therefore, we believe platinum-based drugs are highly likely to promote gene fusion, and future studies could also be devoted to analyzing the aspect of acquired gene fusion resulting from chemotherapeutic agents. At the same time, repeated molecular profiling using comprehensive NGS assays is warranted in NSCLC cases as new targetable drivers like ROS1 can emerge.

Iruplinalkib is a potent inhibitor of ALK kinases approved for ALK-positive NSCLC based on robust responses in the INSPIRE trials in China (9). More encouragingly, multiple trials have confirmed that iruplinalkib is a potent ROS1 inhibitor that inhibits the proliferation and phosphorylation levels of cells expressing ROS 1 fusion proteins (10). In a phase I clinical trial (NCT03389815), it was shown to have antitumor activity and a good safety profile in patients with advanced NSCLC who had ALK/ROS1 rearrangements, with an objective response rate (ORR) value of 44.4% (4/9) for screened patients with ROS1 rearrangements treated with iruplinalkib (11). A range of ROS1/ALK TKIs have been approved for the treatment of patients with ROS1-fusion NSCLC, such as crizotinib, but compared to it, iruplinalkib is less likely to cause liver function abnormalities and diarrhea (11), which is undoubtedly a satisfactory result. This case also exemplifies iruplinalkib’s activity in ROS1-fusion NSCLC patients.

## Conclusion

We present a case of metastatic NSCLC where comprehensive genomic re-testing after multiple systematic lines of therapy revealed a newly emerged ROS1 fusion, not detected at initial diagnosis. Use of the ALK TKI iruplinalkib led to a marked therapeutic response. This highlights the potential for receptor tyrosine kinase fusions like ROS1 to develop as acquired alterations in NSCLC, underscoring the importance of persistent molecular re-profiling to guide appropriate targeted therapy.

## Data Availability

The original contributions presented in the study are included in the article/**Supplementary Material**. Further inquiries can be directed to the corresponding author.

## References

[1] Drilon A Jenkins C Iyer S Schoenfeld A Keddy C Davare MA . ROS1-dependent cancers — biology, diagnostics and therapeutics. Nat Rev Clin Oncol. (2020) 18:35–55. doi: 10.1038/s41571-020-0408-9 32760015 PMC8830365

[2] Drilon A Ou S-HI Cho BC Kim D-W Lee J Lin JJ . Repotrectinib (TPX-0005) is a next-generation ROS1/TRK/ALK inhibitor that potently inhibits ROS1/TRK/ALK solvent- front mutations. Cancer Discov. (2018) 8:1227–36. doi: 10.1158/2159-8290.Cd-18-0484 30093503

[3] Song Z Lu C Xu C-W Zheng Z . Noncanonical gene fusions detected at the DNA level necessitate orthogonal diagnosis methods before targeted therapy. J Thoracic Oncol. (2021) 16:344–8. doi: 10.1016/j.jtho.2020.12.006 33641715

[4] Yang Y Wang Z Fang J Yu Q Han B Cang S . Efficacy and Safety of Sintilimab Plus Pemetrexed and Platinum as First-Line Treatment for Locally Advanced or Metastatic Nonsquamous NSCLC: a Randomized, Double-Blind, Phase 3 Study (Oncology pRogram by InnovENT anti-PD-1-11). J Thoracic Oncol. (2020) 15:1636–46. doi: 10.1016/j.jtho.2020.07.014 32781263

[5] Bai H Wang Z Chen K Zhao J Lee JJ Wang S . Influence of chemotherapy on EGFR mutation status among patients with non–small-cell lung cancer. J Clin Oncol. (2012) 30:3077–83. doi: 10.1200/JCO.2011.39.3744 PMC5321076 22826274

[6] Ren K-H Qin W-W Wang Y Peng J-C Hu W-X . Detection of an EML4-ALK fusion mutation secondary to epidermal growth factor receptor-tyrosine kinase inhibitor (EGFR-TKI) therapy for lung cancer: a case report. Ann Palliat Med. (2022) 11:2503–9. doi: 10.21037/apm-22-744 35927783

[7] Pich O Muiños F Lolkema MP Steeghs N Gonzalez-Perez A Lopez-Bigas N . The mutational footprints of cancer therapies. Nat Genet. (2019) 51:1732–40. doi: 10.1038/s41588-019-0525-5 PMC6887544 31740835

[8] Florea A-M Büsselberg D . Cisplatin as an anti-tumor drug: cellular mechanisms of activity, drug resistance and induced side effects. Cancers. (2011) 3:1351–71. doi: 10.3390/cancers3011351 PMC3756417 24212665

[9] Shi Y Chen J Yang R Wu H Wang Z Yang W . Iruplinalkib (WX-0593) versus crizotinib in ALK TKI-naive locally advanced or metastatic ALK-positive NSCLC: interim analysis of a randomized, open-label, phase 3 study (INSPIRE). J Thoracic Oncol. (2024) 19:912–27. doi: 10.1016/j.jtho.2024.01.013 38280448

[10] Yang Y Zheng Q Wang X Zhao S Huang W Jia L . Iruplinalkib (WX−0593), a novel ALK/ROS1 inhibitor, overcomes crizotinib resistance in preclinical models for non-small cell lung cancer. Investig New Drugs. (2023) 41:254–66. doi: 10.1007/s10637-023-01350-x PMC10140010 37036582

[11] Shi Y Fang J Hao X Zhang S Liu Y Wang L . Safety and activity of WX-0593 (Iruplinalkib) in patients with ALK- or ROS1-rearranged advanced non-small cell lung cancer: a phase 1 dose-escalation and dose-expansion trial. Signal Transduct Target Ther. (2022) 7:25. doi: 10.1038/s41392-021-00841-8 35087031 PMC8795197

