# Supplementary material for: Acquired ROS1 fusion and iruplinalkib response in advanced NSCLC after multiple lines of systematic therapy: a case report
Source: Front Oncol. 2025 Apr 30;15:1571512. doi: 10.3389/fonc.2025.1571512 (PMC12074972; doi:10.3389/fonc.2025.1571512)
Supplement: Supplementary file 1 [file DataSheet1.pdf]

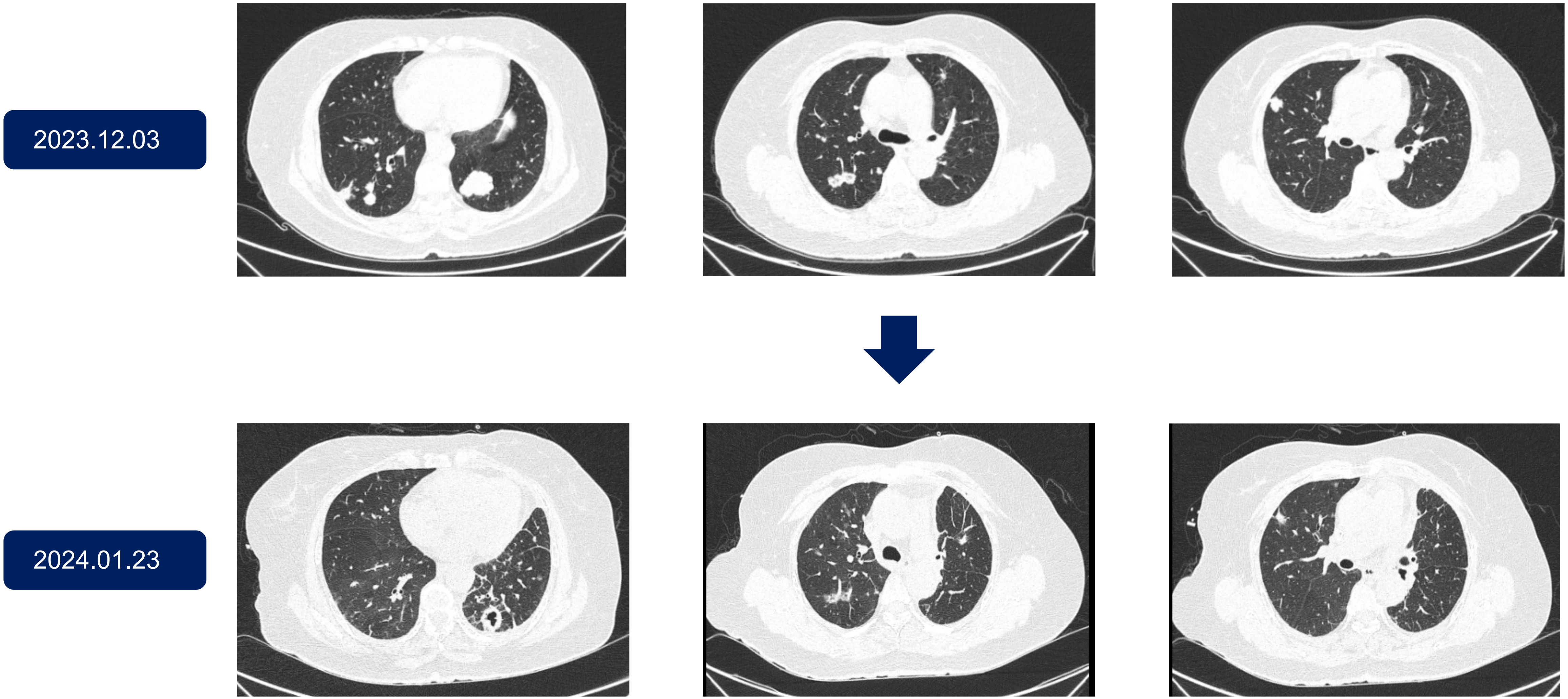

Comparison of CT images before the use of iruplinalkib and after one month of oral administration of iruplinalkib
